# Acute Whole-Body Vibration Exercise Promotes Favorable Handgrip Neuromuscular Modifications in Rheumatoid Arthritis: A Cross-Over Randomized Clinical

**DOI:** 10.1155/2021/9774980

**Published:** 2021-12-02

**Authors:** Ana Carolina Coelho-Oliveira, Ana Cristina Rodrigues Lacerda, Ana Lúcia Cristino de Souza, Luciana Martins de Mello Santos, Sueli Ferreira da Fonseca, Jousielle Márcia dos Santos, Vanessa Gonçalves César Ribeiro, Hércules Ribeiro Leite, Pedro Henrique Scheidt Figueiredo, José Sebastião Cunha Fernandes, Fábio Martins, Renato Guilherme Trede Filho, Mario Bernardo-Filho, Danúbia da Cunha de Sá-Caputo, Alessandro Sartorio, Darryl Cochrane, Vanessa Pereira Lima, Henrique Silveira Costa, Vanessa Amaral Mendonça, Redha Taiar

**Affiliations:** ^1^Centro Integrado de Pós-Graduação e Pesquisa em Saúde (CIPq-Saúde), Universidade Federal dos Vales do Jequitinhonha e Mucuri (UFVJM), Diamantina, Minas Gerais, Brazil; ^2^Programa de Pós-Graduação em Reabilitação e Desempenho Funcional (PPGReab), Universidade Federal dos Vales do Jequitinhonha e Mucuri (UFVJM), Diamantina, Minas Gerais, Brazil; ^3^Faculdade de Ciências Biológicas e da Saúde, Universidade Federal dos Vales do Jequitinhonha e Mucuri (UFVJM), Diamantina, Minas Gerais, Brazil; ^4^Laboratório de Vibrações Mecânicas e Práticas Integrativas, Departamento de Biofísica e Biometria, Instituto de Biologia Roberto Alcântara Gomes e Policlínica Américo Piquet Carneiro, Universidade do Estado do Rio de Janeiro, Rio de Janeiro, RJ, Brazil; ^5^Faculdade de Ciências Agrárias, Universidade Federal dos Vales do Jequitinhonha e Mucuri (UFVJM), Diamantina, Minas Gerais, Brazil; ^6^Istituto Auxologico Italiano, IRCCS, Division of Auxology and Metabolic Diseases, Verbania, Italy; ^7^School of Sport, Exercise & Nutrition, Massey University, New Zealand; ^8^MATIM, Moulin de la Housse, Université de Reims Champagne Ardenne, Reims Cedex 2 51687, France

## Abstract

**Objective:**

Rheumatoid arthritis (RA) causes progressive changes in the musculoskeletal system compromising neuromuscular control especially in the hands. Whole-body vibration (WBV) could be an alternative for the rehabilitation in this population. This study investigated the immediate effect of WBV while in the modified push-up position on neural ratio (NR) in a single session during handgrip strength (HS) in women with stable RA.

**Methods:**

Twenty-one women with RA (diagnosis of disease: ±8 years, erythrocyte sedimentation rate: ±24.8, age: 54± 11 years, BMI: 28 ± 4 kg·m^−2^) received three experimental interventions for five minutes in a randomized and balanced cross-over order: (1) control—seated with hands at rest, (2) sham—push-up position with hands on the vibration platform that remained disconnected, and (3) vibration—push-up position with hands on the vibration platform turned on (45 Hz, 2 mm, 159.73 m·s^−2^). At the baseline and immediately after the three experimental interventions, the HS, the electromyographic records (EMGrms), and range of motion (ROM) of the dominant hand were measured. The NR, i.e., the ratio between EMGrms of the flexor digitorum superficialis (FDS) muscle and HS, was also determined. The lower NR represented the greater neuromuscular efficiency (NE).

**Results:**

The NR was similar at baseline in the three experimental interventions. Despite the nonsignificance of within-interventions (*p* = 0.0611) and interaction effect (*p* = 0.1907), WBV exercise reduced the NR compared with the sham and control (*p* = 0.0003, *F* = 8.86, *η*^2^ = 0.85, power = 1.00).

**Conclusion:**

Acute WBV exercise under the hands promotes neuromuscular modifications during the handgrip of women with stable RA. Thus, acute WBV exercise may be used as a preparatory exercise for the rehabilitation of the hands in this population. This trial is registered with trial registration 2.544.850 (ReBEC-RBR-2n932c).

## 1. Introduction

Rheumatoid arthritis (RA) is a chronic disease that causes progressive damage to the musculoskeletal system. RA compromises neuromuscular control especially in the hands [1–4]. It usually affects the joints in a symmetrical way, thus determining a decline in muscle strength and a progressive reduction of hand functional abilities [5]. Currently, the prevalence of RA is 1-2% of the world's population, with the rate being two to three times higher in women [6] aged between 20 and 65 years [7].

During the pathological process of RA, the individual may have trouble while performing daily tasks induced by pain, stiffness, and deterioration of the joint structure and function. Damage to musculoskeletal tissue caused by RA's inflammation interferes directly with mobility, generation of muscle strength and neuromuscular control [8, 9]. Neuromuscular control is necessary and essential for most daily tasks [10]. Thus, women with RA tend to use higher levels of neuromuscular activation in daily tasks than healthy women [11].

Exercise programs are commonly used to improve hand structure and function, but the disparity in determining the most effective exercise for this population remains inconclusive [11]. Whole-body vibration (WBV) exercise is a neuromuscular stimulus method, which can represent a preparatory exercise in promoting lower joint impact and greater neuromuscular modifications [12].

In the context of rehabilitation of patients with stable RA, it is noteworthy that earlier studies evaluating the effect of WBV focused on the lower limbs [3, 13]. Moreover, few investigations have targeted the upper body by using a static modified push-up position to assess the effects of WBV on healthy participants [14, 15]. Other studies have examined the effect of WBV exposure on neuromuscular activity of the flexor digitorum superficialis (FDS) muscle [15, 16], which is essential for manual skills involving handgrip [17].

Some of the possible mechanisms that may explain the positive effects of WBV exercise are represented by the changes provided in the neuromuscular activation pattern in favor of greater neuromuscular modifications [18]. As evidenced by a two-fold increase in biceps brachial electromyography (EMG) from WBV compared to baseline values [18], it was suggested that this type of treatment can stimulate the neuromuscular system and improve the neuromuscular modifications, i.e., the ratio between EMG and mechanical power, during handgrip activities [19].

From our previous work that reported immediate effects of WBV exercise in promoting a transient increase in muscle contractile performance in untrained healthy women [19, 20], it seems plausible that vibration WBV may be a useful preparatory therapy capable of promoting neuromuscular modifications for the rehabilitation of the hands in women with stable RA. Therefore, the objective of the current study was to investigate the effect of WBV exercise in a single session on the static modified push-up position on the handgrip neuromuscular modifications in women with stable RA. As secondary outcomes, we aimed to evaluate the handgrip strength (HS) and concomitant neuromuscular electrical activity of the FDS muscle and determine if the wrist flexion-extension range of motion (ROM) of the dominant hand was enhanced. It was hypothesized that the acute exposure to WBV directly under the hands would promote an increase in wrist flexion-extension ROM and HS, in addition to a concomitant reduction in electromyography (EMGrms).

## 2. Materials and Methods

### 2.1. Study Design and Participants

**Figure 1:**
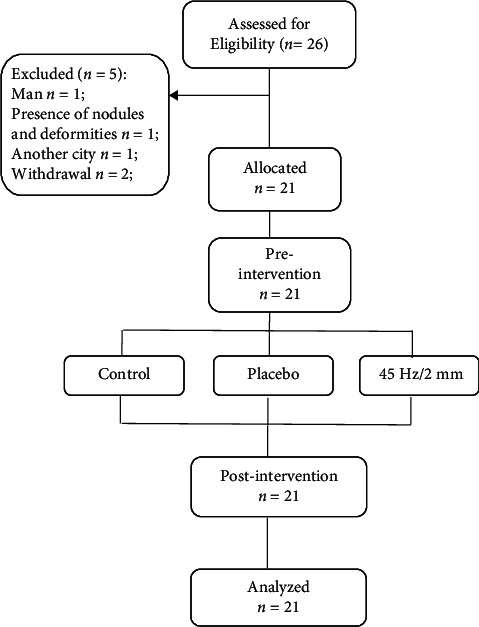
Flow of participants through the study.

Twenty-six women diagnosed with RA were initially screened for eligibility. Twenty-one (*n* = 21) eligible RA women were enrolled the study (Figure 1).

**Figure 2:**
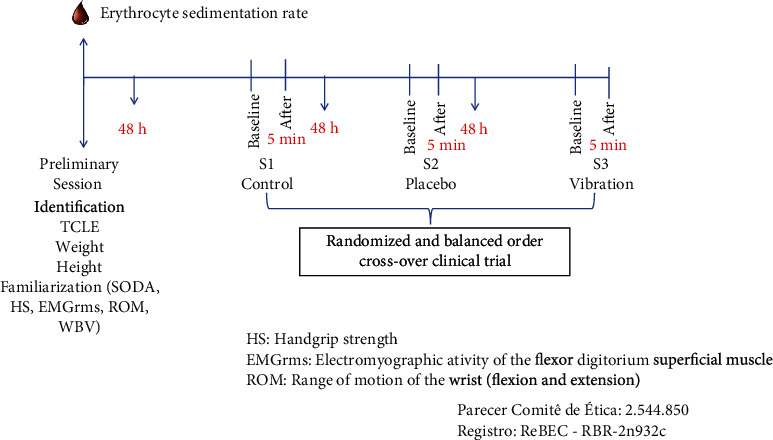
Study design.

The study design was a crossover clinical trial (i.e., all volunteers performed all three experimental interventions in a randomized order) with seven balanced blocks, three by three. Experimental interventions were randomized by sortition, and the participants were blinded. The interventions were performed over a one-week period with a 48-hour recovery following each intervention. The familiarization session was performed 48 hours prior to the beginning of the experimental interventions and included a physical examination, anthropometric measurements (height and body mass), and familiarization with the vibrating platform, HS, EMGrms, and ROM. On the day of familiarization, a blood sample was also performed to check the inflammatory activity of the disease through the erythrocyte sedimentation rate (Figure 2).

This study was conducted in accordance with the checklists for randomized controlled and clinical trials CONSORT and SPIRIT and ethical principles for research involving human subjects (principles of the Declaration of Helsinki). The study received approval from the Ethics Committee of the *Universidade Federal dos Vales do Jequitinhonha e Mucuri* (No. 2.544.850) and was submitted to the Registry of Clinical Trials (ReBEC) (RBR-2n932c).

The participants were recruited between March of 2018 and May of 2019, at the medical clinic by the rheumatologists of the Regional Polyclinic, Basic Health Units and radio advertising in Diamantina, MG, Brazil. The inclusion criteria were as follows: women aged between 20 and 70 years, with confirmed diagnosis of RA by a rheumatologist according to the criteria of the American College of Rheumatology [21]. Participants were ineligible, if they presented the following: sensory disturbances; active infections; alcohol or drug abuse; pregnancy or breastfeeding; anticoagulant treatment; any concomitant disease that would prevent the execution of the experimental interventions; any other rheumatological disease; serious complications of RA; nonstable disease; and intra-articular infiltrations or other procedures, such as physical therapy or corrective surgeries, and some contraindication vibrating platform. Participants were advised to avoid adjusting their drug therapy and the use of analgesics for pain.

### 2.2. Intervention

#### 2.2.1. Experimental Interventions

All the volunteers performed the three experimental interventions at the same time of each day, in a controlled thermoneutral environment (means of 22 ± 1°C and 53 ± 2% relative humidity).

**Figure 3:**
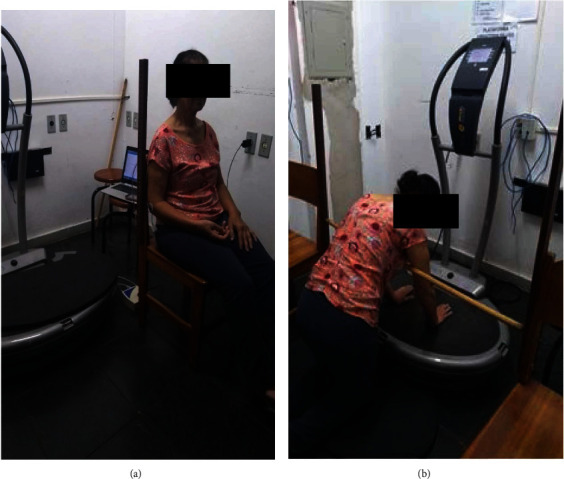
Experimental intervention positions. (a) Control position and (b) push-up position adopted during sham or WBV exercise interventions.

*(1) Control*. Participants remained rested for five minutes in a seated position with feet on the floor and hands in the supine position on the lower limbs. There was no WBV stimulus (Figure 3(a)).

*(2) Sham*. The participants were positioned for five minutes continuously in the push-up position with their hands apart at a distance of 28 cm on the vibrating platform that was disconnected, but with a sonorous stimulus mimicking the WBV (Figure 3(b)).

*(3) Vibration*. The participants were positioned for five minutes continuously in the push-up position with their hands apart at a distance of 28 cm on the vibrating platform turned on, using the vibratory stimulus intensities of 45 Hz, 2 mm, and 159.73 ms^−2^. The vibrating platform (FitVibe, GymnaUniphy NV, Bilzen, Belgium) produced vertical sinusoidal vibrations resulting in a simultaneous and symmetrical movement on both sides of the body during exposure. A horizontal bar at shoulder height was used to avoid trunk flexion during the intervention and to guarantee an elbow flexion of 10° (Figure 3(b)). The WBV parameters of frequency (45 Hz), amplitude (2 mm), and exposure duration of five minutes were selected in accordance with previous studies reporting positive outcomes [19, 20, 22, 23].

### 2.3. Procedures

Prior to all three experimental interventions, each participant rested for fifteen minutes in a seated position with their hands placed in a supine position on the lower limbs. This verified the resting electromyography of the FDS muscle. Thereafter, each participant was positioned in one of the experimental interventions described previously. At baseline and immediately after the intervention, the muscle performance of the dominant hand was evaluated using the HS dynamometer (Jamar, Warrenville, USA). The electromyographic record of the FDS muscle of the dominant hand was simultaneously recorded using a portable electromyography data log instrument (Miotec, Porto Alegre, Brazil). Following the ROM of the wrist, flexion-extension of the dominant hand was measured using a universal goniometer (Fibra Cirúrgica, Joinville, Brazil). All the evaluations were performed by a single blinded researcher.

### 2.4. Outcome Measures

#### 2.4.1. Handgrip Strength (HS)

Participants were seated with feet on the floor, with the arm in adduction and elbow flexed at 90°, forearm in a neutral position, and wrist extension between 0° and 30°. The dominant hand performed three repetitions of 3-second maximum HS. There was a 60-second recovery period between repetitions. HS was determined by the average of the three peak values [24].

#### 2.4.2. Electromyography (EMGrms)

Electromyography of the FDS muscle of the dominant hand was recorded using a one-channel portable electromyography. Two passive Ag/AgCl electrodes (Meditrace, Ludlow Technical Products, Gananoque, Canada) were positioned on the muscle belly of the FDS muscle with a fixed distance of 20 mm, arranged perpendicular to the direction of muscle fibers. One ground electrode was attached to the lateral epicondyle of the humerus according to the position described by SENIAM (Surface Electromyography for the Non-Invasive Assessment of Muscles) [25]. The recorded signals were treated with 10-480 Hz band pass butterworth filters for signal amplitude analysis and to avoid noise. The analog-to-digital conversion of the signals was performed with a 14-bit input A/D hardware resolution, sampling frequency of 2 kHz, common rejection module greater than 100 dB, signal-to-noise ratio less than 3 *μ*V, and system impedance of 109 Ohms. The signal was captured by surface-active differential sensors and recorded as the Root Mean Square (RMS); this is a quantitative indicator in the recruitment of motor units, in *μ*V and the mean frequency in Hz [26]. The electromyography signals were collected in *μ*V, normalized by peak (peak-to-peak) and transformed into % RMS by software (MiotecSuite 1.0.1065) for data analysis [25].

Both HS and EMGrms were determined concomitantly by the average of the three repetitions performed before and after the experimental interventions. The HS frequency was 3000 ms (i.e., 3 s) with the EMGrms analyzed using a sliding window of within the interval range of 1000-2000 ms.

#### 2.4.3. Neural Ratio (NR)

NR was calculated from the EMGrms of FDS divided by the mechanical power (HS). A lower NR represented greater neuromuscular modification [18].

#### 2.4.4. Range of Motion (ROM)

The ROM was measured in degrees from a universal manual goniometer, by a trained researcher. The fixed arm of the goniometer was placed parallel to the longitudinal axis of the proximal end. The movable arm was positioned parallel to the longitudinal axis of the distal end, with the fulcrum at the axis of the joint. Measurements were made of wrist flexion and extension of the dominant hand [27].

## 3. Data Analysis

Data were reported as the mean ± 95%confidence intervals (CIs). Intraclass correlation coefficients assessed the test-retest reliability of the HS and electromyography measures. Shapiro-Wilk's test determined normality, and Levene for homogeneity revealed that the data was normally distributed and homogeneous.

The effects of the interventions were compared by split-plot arrangement in a randomized block design and Tukey's (statistical significance level was set at 5%) test for means comparison (within-test, between-test, and interaction). Thus, the within-test column represented the time factor. The between-test column represented the intervention factor. The interaction column represented the interaction (time x intervention). The effect size (eta squared: *η*^2^) were based on the following criteria: <0.25 represented small effect; between 0.25-0.4, moderate effect; and >0.4, large effect [28].

### 3.1. Sample Size

The sample size was calculated using the G-Power® software (Franz Faul, Universitat Kiel, Germany). A sample size of eighteen participants was required for an error probability set at 5%, a power of 80%, and an effect size of 0.64 this was obtained from a previous work evaluating the dose-response of acute WBV exercise in the push-up position on neural ratio in untrained healthy women [19]. Nevertheless, we considered an attrition rate of 15%; the sample size had twenty-one participants (7 blocks of 3 × 3 participants). There was no participant dropout; therefore, it was not necessary to analyze the data by intention to treat.

## 4. Results

The ICC test-retest reliability of HS, EMG activity of the FDS muscle, and ROM were 0.984, 0.778, and 0.899, respectively.

### 4.1. Characteristics of Participants

Table 1 presents the volunteers characteristics concerning age, anthropometric parameters, identification of the medical diagnosis's period, and erythrocyte sedimentation rate test to verify the inflammatory activity disease.

### 4.2. Primary Outcome

#### 4.2.1. NR

**Figure 4:**
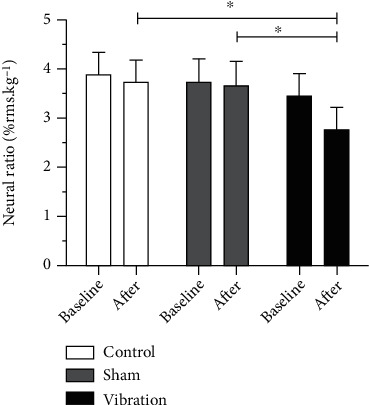
Neural ratio at baseline and after the experimental interventions. ^∗^*p* < 0.05.

NR was similar in the three experimental interventions at baseline [baseline-sham: 3.68 (95% CI: 2.67-4.68), control: 3.88 (95% CI: 2.91-4.85), and vibration: 3.42 (95% CI: 41-4.43)]. Despite no within-interventions (*p* = 0.0611, *F* = 3.94, *η*^2^ = 0.66, power = 0.99) and interaction (*p* = 0.1907, *F* = 1.69, *η*^2^ = 0.50, power = 0.96) effect, between-interventions analyses (*p* = 0.0003, *F* = 8.86, *η*^2^ = 0.85, power = 1.00) showed that acute WBV exercise reduced the NR compared with the sham and control [after-sham: 3.63 (95% CI: 2.58-4.67), control: 3.71 (95% CI: 2.74-4.68), and vibration: 2.74 (95% CI: 1.79-3.69)] (Figure 4).

### 4.3. Secondary Outcomes

#### 4.3.1. HS

HS was similar in the three experimental interventions at baseline. Despite no between-interventions effect, there was interaction effect and within-interventions analyses showed that immediately after the WBV exercise, the HS augmented was approximately 11% compared with the baseline and other experimental interventions (sham and control) (Table 2).

#### 4.3.2. EMGrms

EMGrms was similar in the three experimental interventions at baseline. Despite no within-interventions and interaction effect, the between-interventions analyses demonstrated that acute WBV exercise decreased significantly the EMGrms activity of FDS muscle compared with sham and control (Table 2).

#### 4.3.3. ROM

Wrist flexion-extension ROM was similar in the three experimental interventions at baseline. Within and between-interventions and interaction analyses showed that acute WBV exercise increased both wrist ROM compared with baseline and the other experimental interventions (sham and control) (Table 2).

## 5. Discussion

The current findings suggest that acute WBV exercise directly under the hands promotes favorable handgrip neuromuscular modifications in women with stable RA. This is in agreement with de Souza et al. [19], who reported that in healthy individuals, the push-up position performed on WBV machine promoted an acute positive effect on HS accompanied by a lower index of neural efficiency, providing a better efficiency of muscle contraction. Thus, WBV represents a possibility of preparatory activity with immediate effect to be used prior to rehabilitation session of stable RA patients. Therefore, to understand the neuromuscular modifications provided by this exercise in this population, first, the effects of acute WBV exercise on HS muscular performance concomitant with the neuromuscular activity of the FDS muscles require consideration.

The HS of the current participants was 20.03 kg at baseline in all experimental interventions, representing 62% of the predicted HS in middle-aged and elderly Brazilians. Thus, the disease resulted in a 33 to 37% impact on HS [29, 30]. Considering the measurement properties of the HS assessment, the minimum clinically important difference (MCID) scores for women with carpometacarpal osteoarthritis, a chronic disease which results in deterioration of the joint surfaces bone reformation such as RA, are approximately 0.84 kg (affected side) and 1.12 kg (unaffected side) [30, 31]. Although the MCID was estimated for another chronic disease group, the score obtained in our study was 2.39 kg (1.88–2.90) with WBV, suggesting an important clinical change. This is in agreement with Brorsson et al. [11], who found that patients suffering from arthritis are weaker than healthy individuals in terms of flexion-extension strength. However, our results, as well as those of Villafañe et al. [31] and Speed and Campbell [32], showed that increases of muscle strength in individuals with RA may be due to neural adaptation and, consequently, greater efficiency elicits motor unit activation. Experiments with surface electromyography showed that women with RA tend to use higher levels of neuromuscular activation in daily tasks than healthy women [11], especially during manual skills involving handgrip. In the present study, we opted to evaluate the EMGrms of the FDS muscle. This decision was based on the major muscle group which is responsible for handshake activity since it helps to provide balance for the finger flexion arc [15, 16, 33]. Moreover, we decided for the static push-up position on the vibration platform, as there is evidence of greater muscle activation in the upper limb muscles during acute WBV stimulation of this position [34]. However, in our study, the acute WBV stimulation reduced muscle activation levels immediately after the WBV intervention, suggesting that fewer motor units were required to perform the same handgrip activity.

According to our findings, a single acute WBV exercise session directly under the hands, in a modified static push-up position, was able to promote neuromuscular changes in handgrip in women with RA. We observed that the NR of the participants was about 3.66% at the baseline in all experimental interventions. After exposure to the vibration intervention, there was a reduction of approximately 24.5% in handgrip NR compared to sham and control interventions. These results demonstrate that a single session of acute WBV, directly under the hands, promotes greater neuromuscular modifications. Corroborating the results of de Souza et al. [19], which suggested an acute dose-dependent WBV stimulus in a static push-up position potentiates handgrip myogenic response; additionally, the mechanism underlying this positive effect seems to be related to the stimulation of the neuromuscular system and subsequent postactivation potentiation, leading to neural enhancement.

Although there are few publications in the context of the rehabilitation of patients with stable RA involving WBV and upper limbs. It is noteworthy that studies generally evaluated the effect of training with WBV, focused on the lower limbs [3, 13, 35]. Regarding the changes and consequent neuromuscular modifications of the hands, to our knowledge, there are no studies that have reported this in RA population. However, Krol et al. [34] and other researches demonstrated an increase in the neuromuscular efficiency and concluded that vibration exercise can be useful to stimulate the neuromuscular system in healthy population [14, 19, 20, 36].

The current findings support the concept that the acute WBV exercise potentiates the neural response. The following protocol description reproduces information already reported elsewhere [15, 19]. WBV exercise is reported to represent an alternative exercise for the treatment of RA due to its ability in promoting lower joint impact and greater neuromuscular modifications. Previous studies have reported satisfactory results of using WBV training that ranged from 24 Hz, 2 mm, acceleration [45.43 m·s^−2^] to 30 Hz, 3 mm, acceleration [106.48 m·s^−2^]. Moreover, the stimulus duration varied from 10.5 minutes to 15 minutes intermittently [13, 35, 36]. Nevertheless, the current WBV parameters (frequency: 45 Hz; amplitude: 2 mm, acceleration: 159.73 m·s^−2^) of 5 minutes continuous WBV were selected based on previous research [19, 20, 22, 23]. Additionally, all participants adhered to the 5-minute protocol and successfully completed the vibration without discomfort.

In the context of joint damage in patients with RA, one of the most affected is the wrist (78%) [37]. Thus, usually, a hand's joints present reduction in muscle contraction, firing rate of motor units, ROM, and mobility, as well as change in the muscle fiber type [38]. In the current study, the wrist flexion ROM in the RA group was on average 11.8° lower than normal values [27], and after acute WBV exposure, there was an increase around 4.29° (compared with a sham test showing an increase around 3.39°). Regarding the wrist extension ROM, the RA group presented a 19.4° value lower than the predicted values [27], which increased after acute WBV (compared with sham test showing an increase around 4.15°).

The rationale for WBV exercise as a preparatory activity before training or rehabilitation sessions is based on the premise of promoting “active muscle warm-up” [22, 23]. Active warm-up consists of low-intensity movements that are effective in raising body temperature, promoting tissue warm-up, and producing a variety of improvements in physiological functions [39]. Therefore, warm-up activities are necessary to prepare the body for vigorous physical activity since they increase performance and decrease the risk of muscle injury. Moderate intensity of active warming and passive warming can increase muscle performance by 3 to 9% [39]. In addition, WBV exercise is purported to increase neuromuscular spindle activity, triggering a reflex-stretch response [40], and consequently creates a small and rapid change in muscle length [41].

In the literature, few studies have investigated the effects of acute WBV on ROM that have focused mainly on the lower limb flexibility [42, 43]. According to Oliveira et al. [44], joint ROM is related to functionality and is a determinant factor of morbidity and a mortality predictor in RA patients. Thus, we considered relevant to investigate the effect of acute WBV exercise applied directly to the hand on the ROM of the wrist flexion-extension. The current data suggests that vibratory exercise significantly improved the wrist ROM, probably triggering small and rapid modifications in muscle length. However, this requires further investigation to substantiate this proposition.

Inevitably, this study had some inherent limitations. As this investigation was only performed with RA women, a certain degree of caution should be acknowledged. However, the statistical analyses demonstrated a large effect size within-between-interventions, as well as interaction for NR. The blood analysis of Erythrocyte Sedimentation Rate and the SODA instrument demonstrated that the studied population was not in the inflammatory activity phase of the disease and presented satisfactory manual dexterity. Moreover, because specific conditions were evaluated, such as platform position, stimulus duration, frequency, and amplitude, and EMGrms analyses of only one muscle group; therefore, the findings of this study cannot be extrapolated to other parameters of acute WBV and cannot be generalized to another population.

## 6. Conclusions

In conclusion, acute WBV exercise, directly under the hands, in the push up position, promotes neuromuscular modifications, suggesting positive impact on neuromuscular performance and wrist ROM, with concomitant reduction in handgrip NR in women with stable RA. As clinical relevance, acute WBV exercise under the hands of stable RA patients suggests positive effects on aspects of structure and function related to manual activities that involve object manipulation. Thus, acute WBV exercise may be a complementary and alternative preparatory exercise for the treatment of patients with musculoskeletal dysfunction.

## Figures and Tables

**Table 1:** Characteristics of participants.

Characteristic (*n* = 21)	Mean (95% CI)
Age (yr)	54 (48.99-59.01)
Body mass (kg)	72.9 (66.98-78.82)
Height (m)	1.59 (1.56-1.62)
BMI^∗^ (kg/m^2^)	28.6 (26.69-30.51)
Diagnostic time (yr)	8 (5.36-10.64)
Erythrocyte sedimentation rate (mm/h)	24.8 (18.43-31.17)
SODA^∗^	106 (104.97-107.03)

BMI^∗^: Body Mass Index; SODA^∗^: Sequential Occupational Dexterity Assessment for Patients with Rheumatoid Arthritis.

**Table 2:** Effect of acute whole-body vibration exercise on handgrip strength, electromyographic records of the flexor digitorum superficialis muscle, and wrist range of motion (*n* = 21).

Variable	Control	Sham	Vibration	Within-interventions				Between-interventions				Interaction			
				*p*	*F*	*η* ^2^	Power	*p*	*F*	*η* ^2^	Power	*p*	*F*	*η* ^2^	Power
*HS*															
Baseline (kg)	20.03 (16.52-23.54)	20.68 (17.29-24.07)	19.38 (16.18-22.58)	0.0014	13.70	0.86	1.00	0.3667	1.02	0.37	0.82	0.0178	4.24	0.73	0.99
After (kg)	20.45 (16.79-24.11)	20.95 (17.40-24.50)	21.77 (18.06-25.48)^∗^^#^												
*EMGrms*															
Baseline (% rms)	64.53 (57.77-71.29)	62.82 (56.83-62.81)	53.84 (46.93-60.79)	0.1821	1.91	0.34	0.76	0.0002	9.29	0.87	1.00	0.7403	0.30	0.17	0.39
After (% rms)	62.86 (55.64-70.08)	62.76 (56.81-68.71)	48.92 (39.34-58.50)^#^												
*FLEXION ROM*															
Baseline (°)	79.48 (75.70-83.26)	77.71 (73.58-81.84)	77.28 (73.36-81.20)	0.0059	9.48	0.77	0.99	0.6424	0.44	0.22	0.49	0.0086	5.05	0.76	0.99
After (°)	77.67 (73.62-81.72)	81.10 (77.17-85.03)	81.57 (76.91-86.23)^∗^^#^												
*Extension ROM*															
Baseline (°)	49.00 (41.37-56.63)	52.71 (44.50-60.92)	50.10 (42.86-57.34)	0.0064	9.28	0.84	1.00	0.0218	4.01	0.62	0.99	0.0404	3.34	0.58	0.99
After (°)	51.86 (46.19-57.53)	53.95 (45.18-62.72)	58.10 (51.85-64.35)^∗^^#^												

HS: handgrip strength; EMGrms: electromyographic records; ROM: range of motion; whole-body vibration of 45 Hz/2 mm. Measures performed at baseline and after the experimental interventions. Data are presented as mean (95% confidence interval), *F* values, and eta partial *η*^2^. *N* = 21 subjects in each experimental test. ∗ represents the difference (*p* < 0.05) compared to baseline. # represents the difference (*p* < 0.05) between the interventions (after). There was no difference between the interventions at baseline.

## Data Availability

The datasets used and/or analyzed during the current study are available from the corresponding author on reasonable request.
